# Knockdown of PSMC2 contributes to suppression of cholangiocarcinoma development by regulating CDK1

**DOI:** 10.18632/aging.203463

**Published:** 2021-09-09

**Authors:** Xiaohui Duan, Jianhui Yang, Bo Jiang, Wenbin Duan, Rongguang Wei, Hui Zhang, Xianhai Mao

**Affiliations:** 1 Department of Hepatobiliary Surgery, Hunan Provincial People’s Hospital, The First Affiliated Hospital of Hunan Normal University, Changsha, China; 2 Research Laboratory of Hepatobiliary Tumor, Hunan Provincial People’s Hospital, The First Affiliated Hospital of Hunan Normal University, Changsha, China; 3 Clinical Medical Research Center for Biliary Disease of Hunan Province, Changsha, China; 4 Laboratory of Hepatobiliary Molecular Oncology, Hunan Provincial People’s Hospital, The First Affiliated Hospital of Hunan Normal University, Changsha, China

**Keywords:** cholangiocarcinoma, PSMC2, CDK1, cell proliferation, cell migration

## Abstract

Cholangiocarcinoma (CCA) has been well known as the second most common primary tumor of hepatobiliary system. PSMC2 (proteasome 26S subunit ATPase 2) is a key member of the 19S regulatory subunit of 26S proteasome, responsible for catalyzing the unfolding and translocation of substrates into the 20S proteasome, whose role in CCA is totally unknown. In this study, the results of immunohistochemistry analysis showed the upregulation of PSMC2 in CCA tissues compared with normal tissues, which was statistically analyzed to be associated with CCA tumor grade. Subsequently, the loss-of-function study suggested that knockdown of PSMC2 significantly suppressed cell proliferation, cell migration, promoted cell apoptosis and arrested cell cycle distribution *in vitro*. The decreased tumorigenicity of CCA cells with PSMC2 knockdown was confirmed *in vivo* by using mice xenograft model. In PSMC2 knockdown cells, pro-apoptotic protein Caspase3 was upregulated; anti-apoptotic proteins such as Bcl-2 and IGF-II were downregulated; among EMT markers, E-cadherin was upregulated while N-cadherin and Vimentin were downregulated, by which may PSMC2 regulates cell apoptosis and migration. Furthermore, through RNA-seq and verification by qPCR, western blotting and co-IP assays, CDK1 was identified as the potential downstream of PSMC2 mediated regulation of CCA. PSMC2 and CDK1 showed mutual regulation effects on expression level of each other. Knockdown of PSMC2 could aggregate the influence of CDK1 knockdown on cellular functions of CCA cells. In summary, our findings suggested that PSMC2 possesses oncogene-like functions in the development and progression of CCA through regulating CDK1, which may be used as an effective therapeutic target in CCA treatment.

## INTRODUCTION

Cholangiocarcinoma (CCA) is a kind of highly malignant tumor originated from bile duct epithelial cells, accounting for about 3% of digestive system tumors [1–3]. As the second most common primary tumor of hepatobiliary system, the high invasion of CCA makes it possible to infiltrate or metastasize in the early stage, which leads to a continuous increasing morbidity and mortality in recent years, and the extreme poor prognosis of patients [3]. Although the current research has revealed some potential risk factors closely related to the incidence of CCA, such as papillomatosis of the bile duct, cirrhosis, viral hepatitis, choledochal cyst, chronic hepatolithiasis and so on, the research on the molecular mechanism of CCA is still very insufficient [4]. Up to now, surgical resection is still the preferred choice for the treatment of CCA, and radical surgical resection (including liver transplantation) is the only effective method for curing CCA [2, 4, 5]. In recent years, the proposal of the concept of targeted drugs and the emergence of a large number of molecular targeted drugs have brought a revolution to the treatment of malignant tumors, which has been playing a brilliant role in the treatment of a variety of tumors [5–7]. However, due to the obscure understanding of the molecular mechanism of CCA, there is no specific targeted drug for CCA in clinical use [8–10]. Therefore, seeking the key regulatory factors in the occurrence and development of CCA and utilizing them as the target of CCA treatment can lay a solid foundation for changing the treatment mode and efficiency of CCA in the future.

The degradation of cellular proteins mediated by the ubiquitin-26S proteasome pathway is a complex and rigorous process in eukaryotic cells. This highly selective protein degradation pathway plays an important role in the regulation of cell cycle progression [11], apoptosis [12], metabolic regulation [13], signal transduction [14] and so on. As far as current knowledge is concerned, 26S proteasome is a multiple-subunit protein complex composed of the 20S degradation complex (CP) and the 19S regulatory complex (RP) [15]. Also, PSMC2 (proteasome 26S subunit ATPase 2), as an essential member of the 19S regulatory subunit, is mainly engaged in catalyzing substrates and transporting them into CP for degradation including various types of cellular proteins, such as the cell cycle protein, cell apoptosis protein, signal transcription and DNA repair protein [16]. What’s more, altered regulation of these cellular proteins is linked to the development and progression of cancer [17]. Based on the pivotal roles played by 26S proteasome in biological processes, there has been an increasing interest in the potential of 26S proteasome as a promising target for various cancer targeted therapies [16]. For example, the previous research reported that PSMC2 depletion in tumor cells could control tumor growth, thereby PSMC2 was considered as a potential gene for the treatment of multiple cancers [17]. In addition, the results from another report demonstrated that the higher expression of PSMC2 correlated with poorer survival rate of patients with CRC. The silencing of PSMC2 had a significant effect on colorectal cancer cells including suppressing cell proliferation and migration, and inducing cell apoptosis [18]. Despite that PSMC2 is identified as a newly discovered cancer-related gene, little is concerning the expressional correlation and functional importance of PSMC2 in CCA.

In this study, the expression pattern of PSMC2 in CCA was revealed through immunohistochemistry analysis, showing that: 1) expression of PSMC2 in CCA tissues is observable higher than normal tissues; 2) CCA tissues with advanced malignant grade tend to express higher PSMC2 level. More evidence proving the promotion of CCA by PSMC2 was provided by the subsequent functional investigations, which exhibited that knockdown of PSMC2 could disturb the proliferation and migration ability of CCA cells while facilitating cell apoptosis by regulating apoptosis or epithelial-mesenchymal transition (EMT) related proteins. Moreover, the inhibited tumorigenicity of CCA cells by PSMC2 knockdown was also manifested *in vitro* by colony formation assay and *in vivo* by mice xenograft model. The exploration of downstream mechanism further recognized the involvement of CDK1, which is a critical regulatory factor in cell cycle, in PSMC2-induced promotion of CCA. These results indicated the essential role of PSMC2 in the development of CCA, which may act as an effective therapeutic target in the treatment of CCA.

## RESULTS

### PSMC2 was upregulated in CCA tissues and expressed in CCA cells

**Figure 1:**
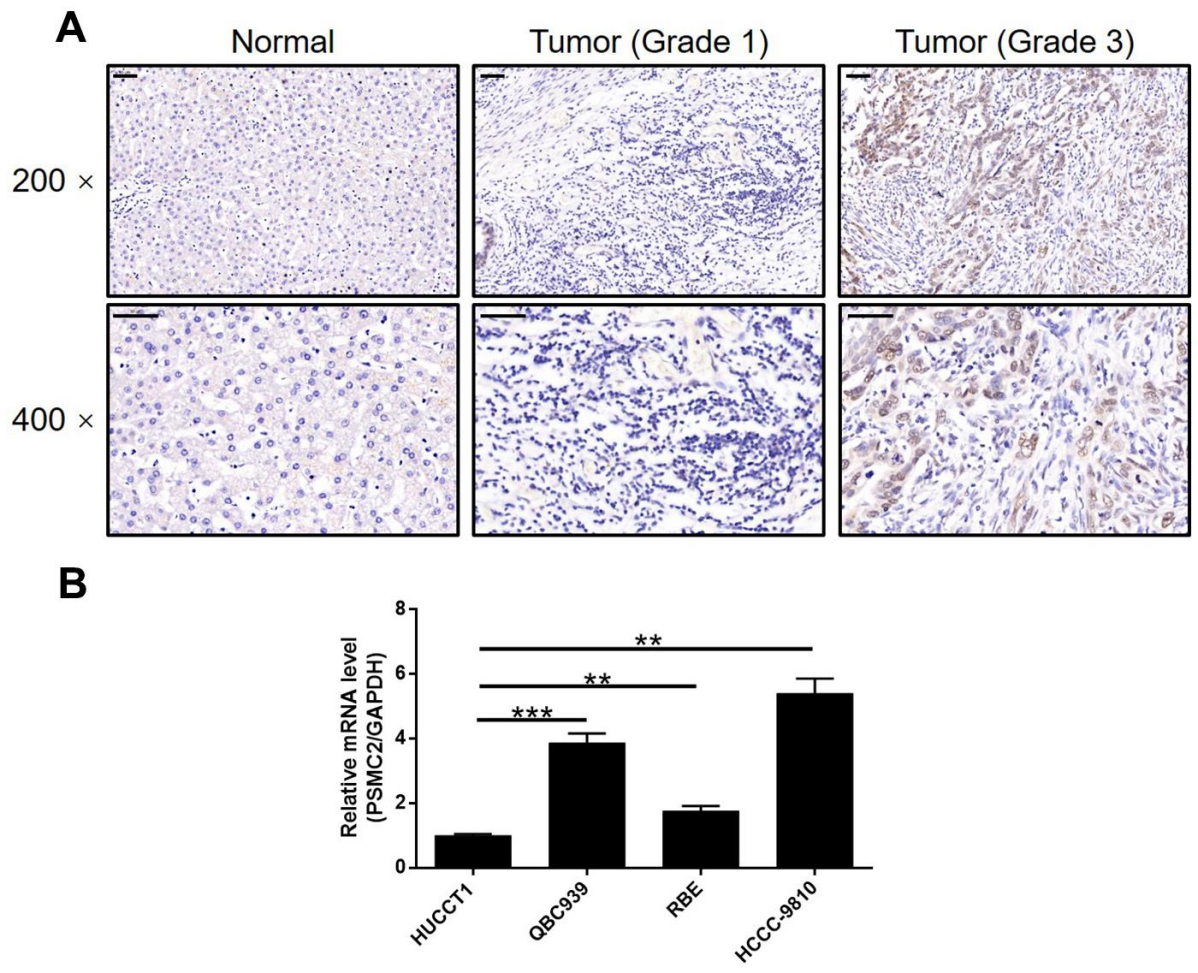
**PSMC2 was upregulated in CCA tissues and abundantly expressed in CCA cells.** (**A**) The expression level of PSMC2 was detected by IHC analysis in CCA tissues and normal tissues (scale bar = 50 μm). (**B**) The endogenous expression of PSMC2 in CCA cell lines including HUCCT1, QBC939, RBE and HCCC-9810 was evaluated by qPCR. Data was shown as mean ± SD. *P < 0.05, **P < 0.01, ***P < 0.001.

IHC was used to map the expression of PSMC2 in tissues from patients with CCA in comparison with tissues from normal controls. These analyses clearly demonstrated that the expression levels of PSMC2 were upregulated in CCA (Figure 1A). Statistical analyses of 74 CCA tissues and 5 normal tissues showed that the expression levels of PSMC2 were significantly higher in the CCA tissues (*P* < 0.001, Table 1). We also performed correlation analysis to investigate the relationship between the expression levels of PSMC2 and specific clinical characteristics. These analyses showed the expression levels of PSMC2 were significantly upregulated in patients suffering from advanced tumor grades (*P* < 0.05, Figure 1A and Table 2). These findings were further validated by Spearman’s rank correlation analysis (Supplementary Table 4). Our analyses demonstrated clear that more serious cases of CCA exhibited higher levels of PSMC2, thus suggesting that PSMC2 may be associated with the progression of CCA. We also determined the endogenous expression of PSMC2 in several CCA cell lines, including HUCCT1, QBC939, RBE, and HCCC-9810. Our analyses showed that QBC939 and HCCC-9810 cells exhibited high expression levels of PSMC2 expression and were therefore selected to construct a cell model of PSMC2 knockdown for subsequent research (Figure 1B).

**Table 1:** Expression patterns of PSMC2 in cholangiocarcinoma tissues and normal tissues revealed in immunohistochemistry analysis.

**PSMC2 expression**	**Tumor tissue**			**Normal tissue**	
	**Cases**	**percentage**		**Cases**	**percentage**
Low	39	52.7%		5	100%
High	35	47.3%		0	-

*P* < 0.001.

**Table 2:** Relationship between PSMC2 expression and tumor characteristics in patients with cholangiocarcinoma.

**Features**	**No. of patients**	**PSMC2 expression**		***P* value**
		**Low**	**High**	
All patients	74	39	35	
Age (years)				0.488
<60	37	21	16	
≥60	37	18	19	
Gender				0.797
Male	39	20	19	
Female	35	19	16	
Grade				<0.001
1	10	9	1	
2	38	25	13	
3	22	1	21	
lymphatic metastasis (N)				0.750
N0	58	30	28	
N1	16	9	7	
T Infiltrate				0.891
T1	6	5	1	
T2	34	15	19	
T3	31	18	13	
T4	3	1	2	

### The depletion of PSMC2 inhibited the development of CCA *in vitro*

**Figure 2:**
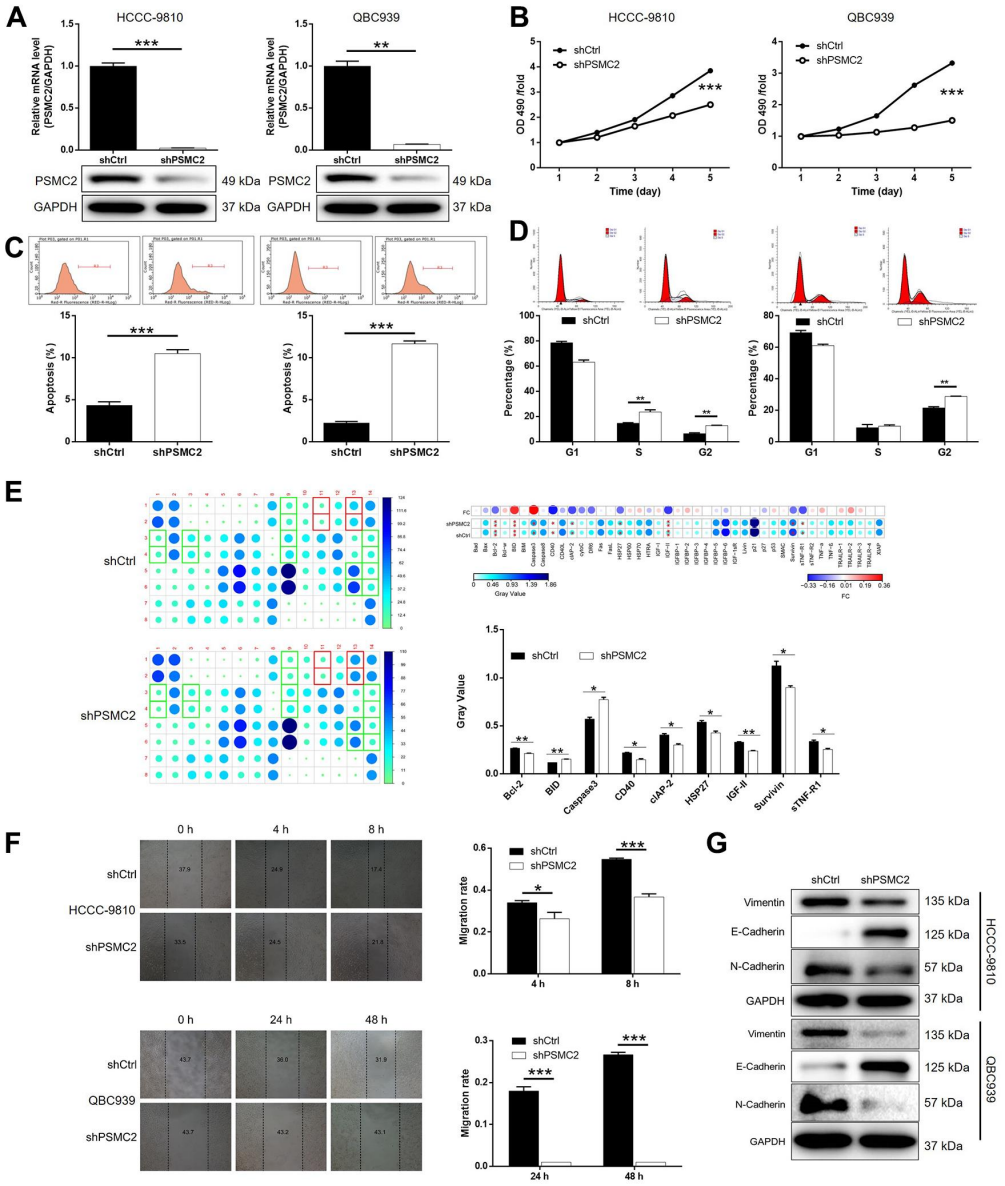
**PSMC2 knockdown inhibited CCA development *in vitro*.** (**A**) Cell models with or without PSMC2 knockdown were constructed by transfecting shPSMC2 or shCtrl. The knockdown efficiency of PSMC2 in HCCC-9810 and QBC939 cells was assessed by qPCR and western blotting. (**B**) MTT assay was employed to show the effects of PSMC2 on cell proliferation of HCCC-9810 and QBC939 cells. (**C**, **D**) Flow cytometry was performed to detect cell apoptosis (**C**) and cell cycle distribution (**D**) of HCCC-9810 and QBC939 cells with or without PSMC2 knockdown. (**E**) Human Apoptosis Antibody Array was utilized to analyze the regulatory ability of PSMC2 on expression of apoptosis-related proteins in HCCC-9810 cells. (**F**) Wound-healing assay was performed to distinguish cell migration of HCCC-9810 and QBC939 cells with or without PSMC2 knockdown. (**G**) WB was used to detect the expression of EMT related proteins in CCA cell models. The representative images were selected from at least 3 independent experiments. Data was shown as mean ± SD. *P < 0.05, **P < 0.01, ***P < 0.001.

We constructed a cell model of PSMC2 deficiency by transfecting a lentivirus that was specifically designed to silence PSMC2. Our intention was to use this model to investigate CCA and the mechanisms that underlie the progression of this disease. Over 80% of the cells tested emitted fluorescence, thus indicating that transfection had been successful (Supplementary Figure 1). qPCR and western blotting were also used to demonstrate that the expression levels of PSMC2 had been downregulated at the mRNA level (*P* < 0.001 for HCCC-9810 cells, *P* < 0.01 for QBC939 cells) and at the protein level (Figure 2A). These data confirmed that the cellular model of PSMC2 knockdown had been successfully created in both HCCC-9810 and QBC939 cells. Furthermore, MTT assays demonstrated that cells in which PSMC2 had been depleted (shPSMC2) grew significantly more slowly than those without PSMC2 depletion (shCtrl) (*P* < 0.001, Figure 2B).

Flow cytometry demonstrated that cell proliferation was influenced by cell apoptosis in CCA cells, either with or without the knockdown of PSMC2. Cells that were deficient in PSMC2 showed a significantly greater proportion of apoptotic cells than the shCtrl group (*P* < 0.001, Figure 2C). Cell cycle analyses further showed that the downregulation of PSMC2 in HCCC-9810 and QBC939 cells was associated with significant cell cycle arrest in the G2 phase (*P* < 0.001, Figure 2D). Next, we used an antibody array to investigate the effect of PSMC2 on apoptosis-related proteins. This array was designed to help us identify the specific mechanisms underlying the ability of PSMC2 to regulate cellular apoptosis. The antibody array demonstrated that PSMC2 knockdown led to an upregulation in the expression levels of BID and caspase 3, and a downregulation in the levels of Bcl-2, CD40, cIAP-2, HSP27, IGF-II, Survivin, and sTNF-R1 (Figure 2E). In addition, wound-healing assays were used to investigate the mobility of HCCC-9810 and QBC939 cells in control and shPSMC2 groups. We observed a significant reduction of cell mobility in the shPSMC2 groups for both cell lines (*P* < 0.001, Figure 2F). Data further suggested that these effects were caused by the upregulation of E-cadherin and the downregulation of both N-cadherin and Vimentin (Figure 2G). Collectively, our findings suggest that PSMC2 may play a key functional role in the development of CCA by regulating cellular migration, colony formation, and apoptosis.

### PSMC2 regulates CCA by targeting CDK1

**Figure 3:**
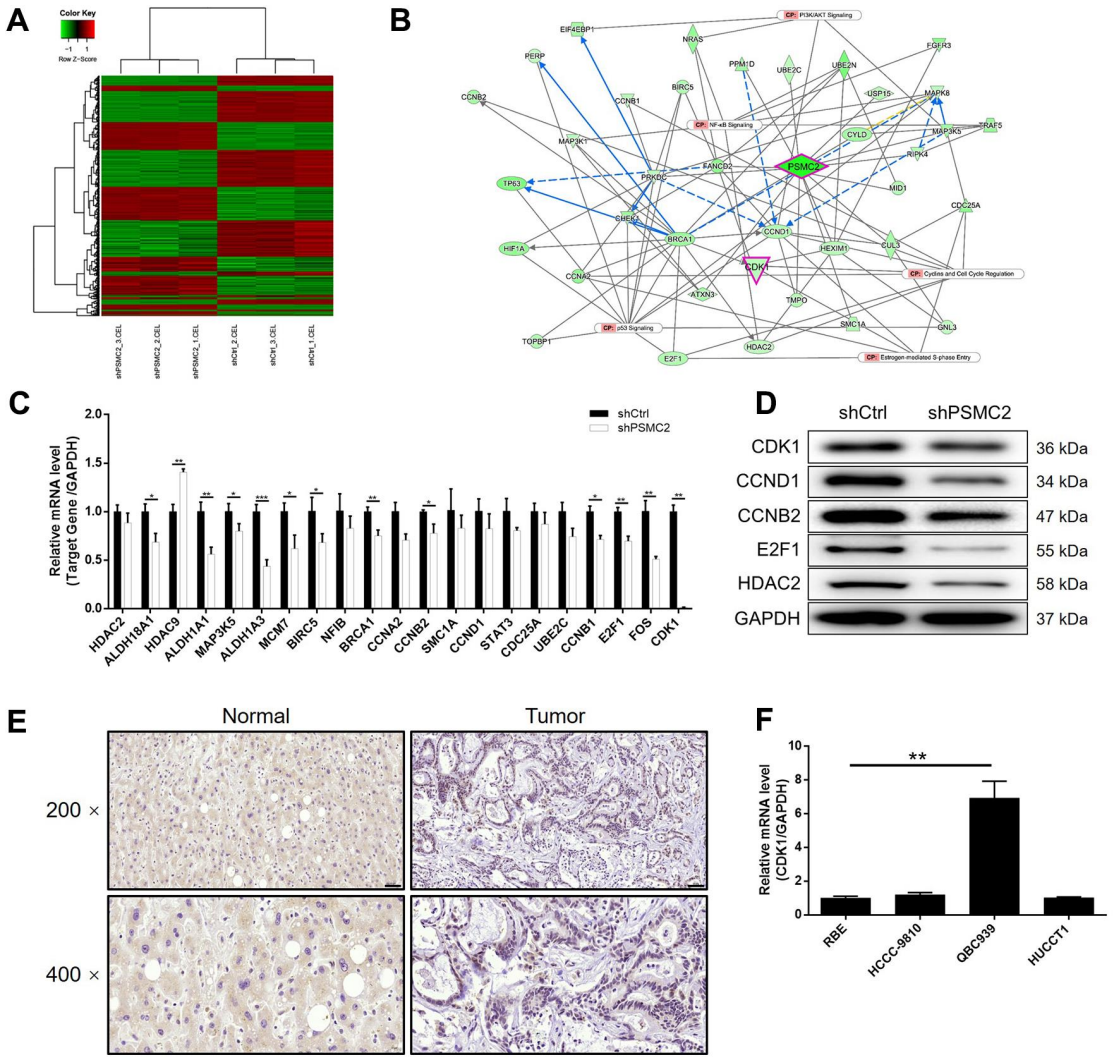
**The exploration and verification of downstream underlying PSMC2 induced regulation of CCA.** (**A**) A PrimeView Human Gene Expression Array was performed to identify the differentially expressed genes (DEGs) between shPSMC2 and shCtrl groups of HCCC-9810 cells. (**B**) A PSMC2-induced interaction network was established based on IPA analysis. qPCR (**C**) and western blotting (**D**) were used to detect the expression of several selected DEGs in HCCC-9810 cells with or without PSMC2 knockdown. (**E**) The expression of CDK1 in CCA tissues and normal tissues was evaluated by IHC analysis (scale bar = 50 μm in 200 magnification, scale bar = 20 μm in 400 magnification). (**F**) The mRNA expression of PSMC2 in CCA cell lines was detected by qPCR. The representative images were selected from at least 3 independent experiments. Data was shown as mean ± SD. *P < 0.05, **P < 0.01, ***P < 0.001.

Prior to this study, we knew very little about how PSMC2 was associated with the regulation of CCA. In the present study, we used 3 v 3 RNA-seq to identify differentially expressed genes (DEGs) between HCCC-9810 cells in the shPSMC2 group and the shCtrl group. We identified 1106 DEGs that were upregulated, and 1261 DEGs that were downregulated, in the shPSMC2 cells when compared with the shCtrl cells (Supplementary Figure 2A and Figure 3A), when using a |Fold Change| ≥ 1.3 and an FDR < 0.05 (the *P* value after Benjamini-Hochberg analysis) as threshold criteria. Next, we used IPA analysis to investigate how the changes in the 2367 DEGs might influence canonical signaling pathways or IPA disease and function (Supplementary Figure 2B, 2C). Based on IPA analysis, and the bioinformatic analysis of a PSMC2 interaction network, we identified several DEGs that showed maximal levels of fold-change; these findings were then verified by qPCR and western blotting in HCCC-9810 cells. The most promising candidate target for PSMC2 was CDK1, a core component in the pathway that regulates cyclin and the cell cycle (Supplementary Figures 2B, 3) (Figure 3B–3D). CDK1 expression levels exhibited a similar profile to those of PSMC2 that expression levels were significantly higher in CCA tissues than in normal control tissues (Figure 3E). qPCR also confirmed the significant upregulation of CDK1 levels in CCA cells (Figure 3F). Collectively, these findings demonstrated that CDK1 may represent a potential target of PSMC2 in the regulation of CCA, although this now needs to be validated *in vitro*.

### The knockdown of PSMC2 aggravated the inhibition of CCA progression induced by the depletion of CDK1

**Figure 4:**
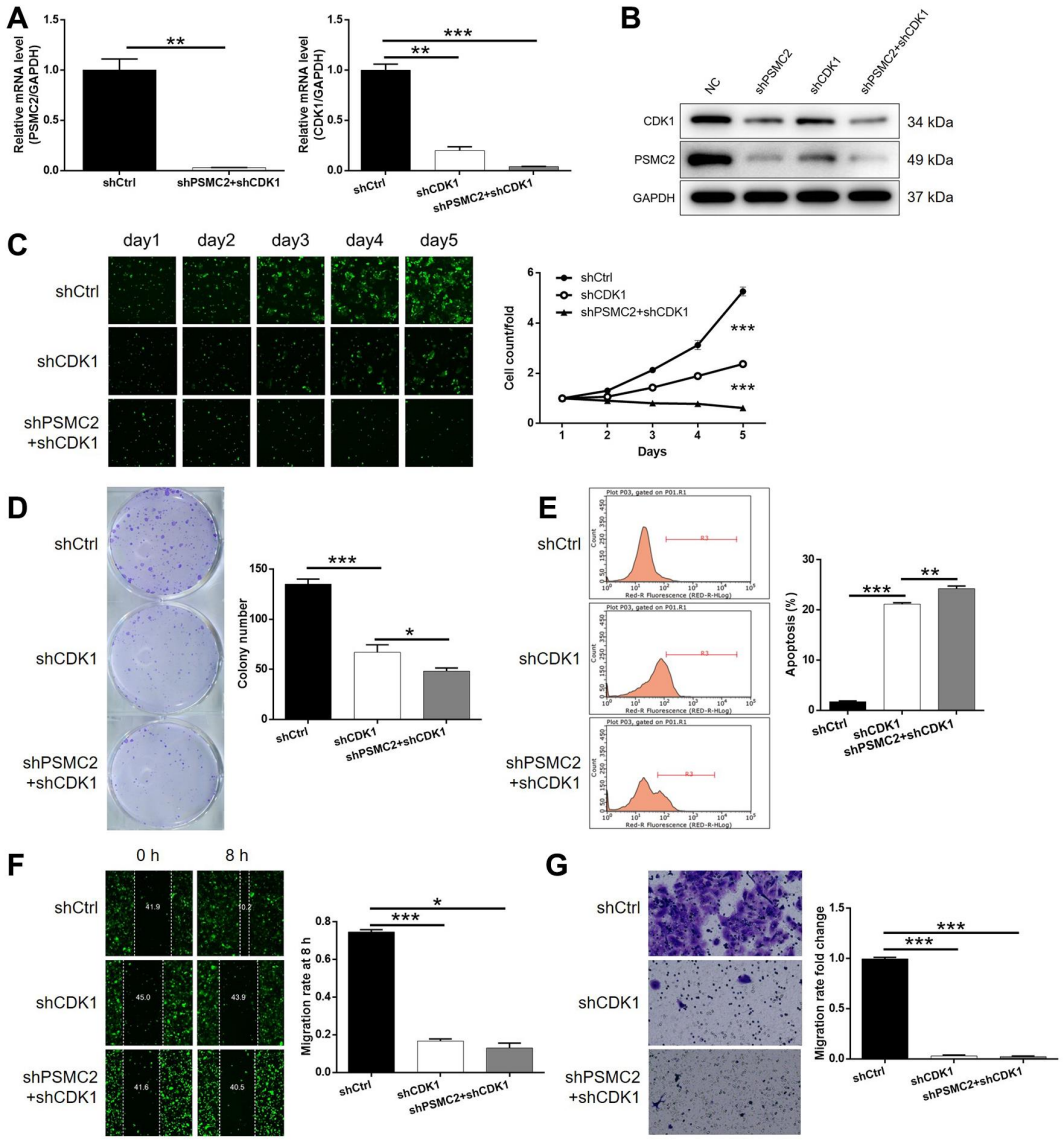
**Knockdown of PSMC2 deepens the effects on CCA cells by CDK1 knockdown.** (**A**, **B**) The expression of PSMC2 and CDK1 in HCCC-9810 cells transfected with shCtrl, shCDK1 and simultaneous shPSMC2 and shCDK1 were detected by qPCR (**A**) and western blotting (**B**). Cell models were subjected to the detection of cell proliferation by Celigo cell counting assay (**C**), colony formation (**D**), cell apoptosis (**E**), cell migration by wound-healing assay (**F**) and Transwell assay (**G**). The representative images were selected from at least 3 independent experiments. Data was shown as mean ± SD. *P < 0.05, **P < 0.01, ***P < 0.001.

Next, we transfected HCCC-9810 cells with shCDK1, or both shPSMC2 and shCDK1, to investigate their combined action in terms of CCA. First, we evaluated the transfection efficiency as described earlier. In brief, we identified the most effective shRNA for silencing CDK1 by performing qPCR (Supplementary Figure 4). As shown in Figure 4A, 4B, it was clear that there was a relationship between the expression levels of PSMC2 and CDK1. The depletion of PSMC2 downregulated the levels of CDK1 while a deficiency in CDK1 led to a downregulation of PSMC2. Our analyses demonstrated that the knockdown of CDK1 exhibited significant effects on cell proliferation, colony formation, and cellular apoptosis (*P* < 0.001, Figure 4C–4E), as also seen with PSMC2. Cell migration may be the main mechanism underlying tumor metastasis and was also investigated. As shown in Figure 4F, 4G, a reduction in CDK1 levels significantly inhibited the cell migration ability of HCCC-9810 cells, as determined by both wound-healing and Transwell assays. We also demonstrated that the additional knockdown of PSMC2 in CDK1 knockdown cells had deleterious effects on a range of cellular functions, including colony formation, cell proliferation, cell apoptosis, and migration (*P* < 0.001, Figure 4C–4G). The combination of CDK1 overexpression and PSMC2 knockdown had no significant effects on cell apoptosis and cell migration in CCA cells (Supplementary Figure 5). Collectively, these results indicated the crucial role of the PSMC2/CDK1 axis in the development of CCA.

### Identifying the mechanisms underlying the suppressive effect of PSMC2 on tumor growth *in vivo*

**Figure 5:**
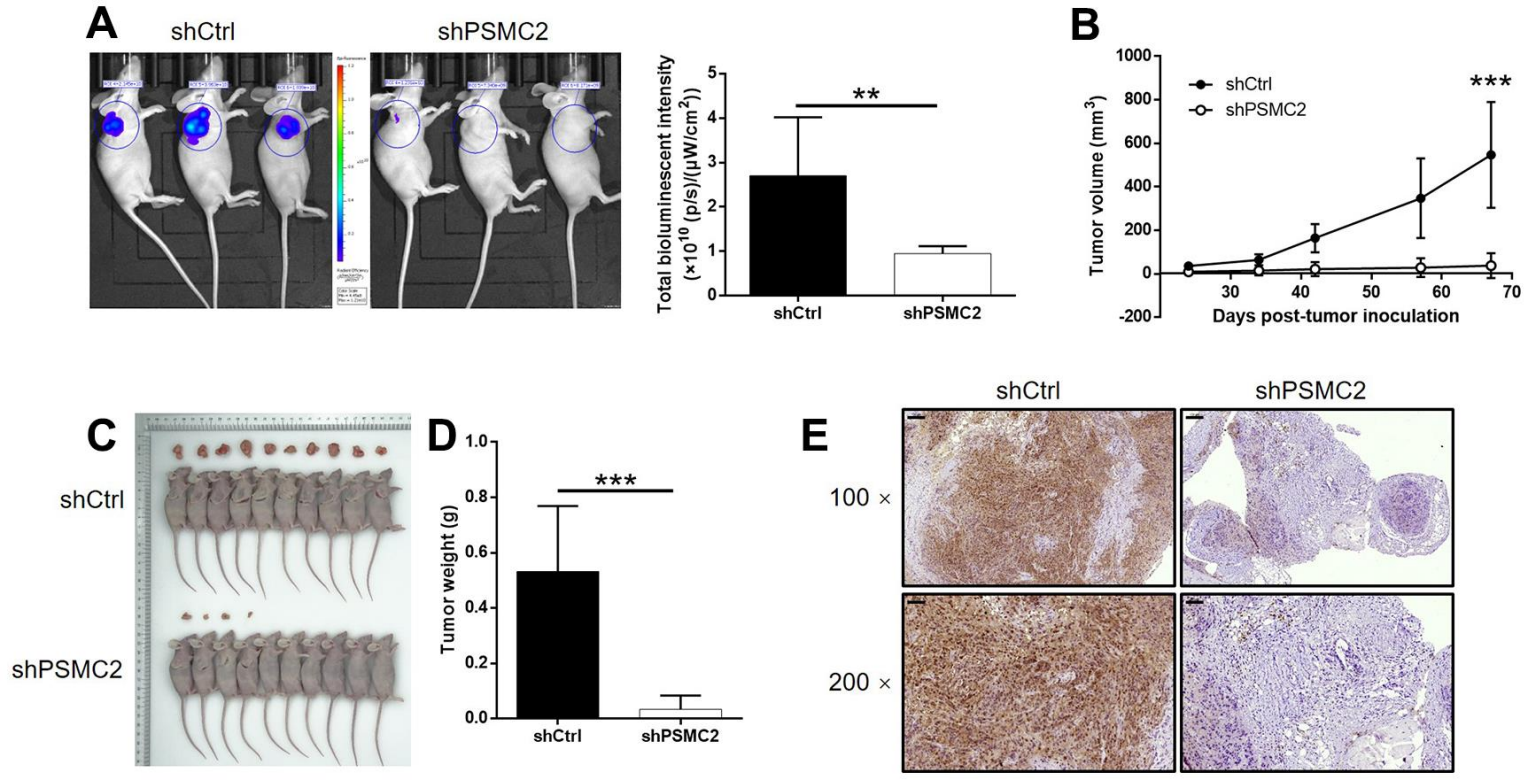
**PSMC2 knockdown inhibited CCA development *in vivo*.** (**A**) *In vivo* imaging was performed to evaluate the tumor burden in mice of shPSMC2 and shCtrl groups at day 67 post tumor-inoculation. The bioluminescence intensity was scanned and used as a representation of tumor burden in mice of shPSMC2 and shCtrl groups. (**B**) 24 days post injection of HCCC-9810 cells with or without PSMC2 knockdown, the volume of tumors formed in mice was measured and calculated at indicated time intervals. (**C**, **D**) Mice were sacrificed at day 67 post injection, and the tumors were removed for collecting photos (**C**) and weighing (**D**). (**E**) After removing the tumors, the Ki67 index was evaluated by IHC staining as a representative of tumor growth activity (scale bar = 100 μm in 100 magnification, scale bar = 50 μm in 200 magnification). Data was shown as mean ± SD. *P < 0.05, **P < 0.01, ***P < 0.001.

We successfully constructed mouse models by injecting HUCCT1 cells (with or without PSMC2 knockdown). Subsequently, *in vivo* bioluminescence imaging demonstrated that the total bioluminescence intensity was significantly weaker in the shPSMC2 group (*P* < 0.001, Figure 5A) and that the tumor burden was significantly smaller. In addition, the solid tumors in the shPSMC2 group had a reduced volume and smaller weight, thus highlighting the suppressive effects of PSMC2 silencing on tumor growth (*P* < 0.001, Figure 5B–5D). Tumors removed from mice in the shPSMC2 group, also had a lower Ki67 index and exhibited lower proliferative activity (Figure 5E).

## DISCUSSION

The contribution of this work has been to confirm the mechanistic roles of PSMC2 in the development and progression of CCA. The behavior of the assessment of PSMC2 expression in tissues specimens made us conclude that compared with normal tissues, the expression level of PSMC2 was much higher in CCA tissues, especially in advanced-grade CCA tissues. One of the more significant findings to emerge from this study was that the cells with low-expression PSMC2 exhibited slower cell proliferation rate, weaker migration ability and arrest of cell cycle in G2 phase, and were prone to apoptosis. Taken together, these results revealed the promoting effect of PSMC2 in CCA. Consistently, the data from *in vivo* experiments were in line with the results of *in vitro* studies, indicating that PSMC2 knockdown exerted its tumor suppressive function in CCA.

The ubiquitin-26S proteasome pathway, an ATP-dependent non-lysosomal protein degradation pathway, can efficiently and highly selectively degrade intracellular proteins, especially short-lived functional proteins and oncogene products. 26S proteasome is composed of 20S core catalytic particles and a variety of regulatory components such as 19S and PA28 regulatory particles. It is generally believed that 19S particles are involved in promoting the degradation of proteins by 20S particles through recognizing the position of ubiquitinated protein substrates and unfolding them [19, 20]. PSMC2 is an indispensable component of 19S subunit, and can bind to ATP and nucleotides. One of its outstanding features is the involvement in the selective degradation of intracellular proteins [21]. Nijhawan et al. reported that PSMC2 obtained the highest ranking among the top 56 candidate CYCLOPS genes based on the characteristics of spliceosome, proteasome and ribosome components, reflecting the essential importance of PSMC2 in cancer cell proliferation or survival [16]. On the other hand, considerable research efforts from Song et al. demonstrated that the down-regulated PSMC2 had inhibition effects on the development and progression of osteosarcoma via suppressing the abilities of cell proliferation, migration and colony formation, arresting the cell cycle in G2/M phase, as well as inducing cell apoptosis [22]. More importantly, another research from Li et al. revealed that miR-630 promoted osteosarcoma cell proliferation, migration and invasion by targeting PSMC2, which provided strong evidence for the potential link between PSMC2 and osteosarcoma [23]. Nevertheless, the interaction between PSMC2 and CCA has not been investigated thoroughly and little is known about the functional roles of PSMC2 in CCA.

For mining the mechanism by which PSMC2 regulates the cell apoptosis, a Human apoptosis antibody array was utilized to visualize the differential expression of apoptosis-related proteins in shCtrl and shPSMC2 groups. For example, the well-known anti-apoptotic protein Bcl-2 and pro-apoptotic BID in Bcl-2 protein family were found to be downregulated and upregulated, respectively [24–26]. Caspase3, an essential participator in cell apoptosis was also indicated to be downregulated in shPSMC2 group of CCA cells [27]. cIAP-2, a member of IAP protein family which possess stronger anti-apoptosis ability than Bcl-2 family, has been reported to have critical functions in a variety of malignancies and was also found to be downregulated by PSMC2 knockdown [28, 29]. Moreover, accumulating evidence has suggested the regulatory function of human cancers by Survivin [30], which is a new member of anti-apoptotic protein family and whose expression was found to be decreased by PSMC2 knockdown in this work. Otherwise, several other factors that has been linked with apoptosis such as CD40 and IGF-II were also detected and proved to be involved. On the other hand, sufficient evidence has been provided to illustrate the critical role of EMT, which is key for development process, in metastasis of tumors [31, 32]. It has been widely acknowledged that downregulated expression of the epithelial cell marker E-cadherin and upregulation of the stromal cell marker N-cadherin are important features of EMT process [33]. Furthermore, Vimentin is an important EMT marker, which has also been demonstrated to influence the development and metastasis of tumors [33, 34]. In this study, the altered expression levels of E-cadherin, N-cadherin and Vimentin suggested that PSMC2 may promote CCA cell migration through affecting EMT process.

Cell cycle progression is the core event of all proliferating cells, which is mainly driven by cyclin dependent kinases (CDKs). CDK1 (also known as Cdc2) is the only cyclin dependent kinase that regulates the cell cycle process in lower organisms, and it plays a role by binding with cyclin in mammals. Although the related research of CDK1 is mainly driven by the exploration of cell cycle regulation, a large number of studies in recent years have shown that CDK1 has a high diversity of functions, especially the important role in the survival of tumor cells [35]. For instance, Ravindran Menon et al. expounded that overexpression of CDK1 could promote the spheroid forming ability, tumorigenic potential, and tumor-initiating capacity of melanoma cells, which was further rationalized by the CDK1-driven transcriptional activation of Sox2 but not its role in cell cycle [36]. Moreover, CDK1 was recently identified as the target of a competing endogenous RNA (ceRNA) mechanism (lncRNA PVT1/miR-31) in the facilitation of bladder cancer progression [37]. Besides, the capability of regulating cell cycle of CDK1 in CCA was also revealed to some extent [38]. Therefore, it may be of guiding significance for tumor therapy to uncover the multifaceted function and mechanism of CDK1 in cells. In this study, CDK1 was proposed as the downstream of PSMC2 in the regulation of CCA. The upregulation of CDK1 in CCA was observed, knockdown of which inhibited CCA development through influencing phenotypes including cell proliferation, colony formation, cell migration and cell apoptosis. More importantly, PSMC2 and CDK1 possessed positive mutual regulation ability to each other. Furthermore, simultaneous knockdown of PSMC2 and CDK1 exhibited stronger inhibition of CCA than mere PSMC2 or CDK1 knockdown; overexpression of CDK1 could partially alleviate PSMC2-induced regulation of CCA.

In summary, our study revealed the essential role of PSMC2 in the development and progression of CCA, which may be executed through the interacting CDK1. Therefore, PSMC2 may act as a tumor promotor in CCA and could be used as a therapeutic target in the treatment of CCA. Despite, this study was still limited by the relatively small amounts of clinical specimens and the ambiguous molecular mechanism, which would be improved in our future work.

## MATERIALS AND METHODS

### Immunohistochemistry (IHC)

Paraffin-embedded cancer/normal tissues microarray chip (Xi’an Alenabio Co., Ltd, China) was applied for immunohistochemistry staining to identify PSMC2 and CDK1. Antigen of the tissue slide chip was retrieved, next slides were blocked by 3% H_2_O_2_ and appropriate serum, then PSMC2 and CDK1 antibodies were incubated with the slides overnight at 4° C. After washing, appropriate second antibody was added and cultured at room temperature for 2 h. After staining, tissues pictures were collected and viewed using ImageScope software and CaseViewer. Antibodies used were detailed in Supplementary Table 1.

### Cell lines and lentiviral vector cell transfection

HCCC-9810, HUCCT1, RBE, and QBC939 cells lines (BeNa Technology, China) were cultured in an incubator 37° C 5% CO_2_ as cell protocols described. shRNAs of human PSMC2 and CDK1 were designed and lentivirus was produced in Shanghai Bioscienceres, Co., Ltd. and Supplementary Table 2 showed the detailed RNA sequences. Cells were transfected with lentivirus and stable expression cells were collected after three days and used for our study.

### qRT-PCR

RNA was isolated using TRIzol (Sigma) and the quality was evaluated by Thermo Nanodrop 2000/2000C spectrophotometer. M-MLV RT kit (Promega) was used to obtain cDNA and SYBR Green Master Mix Kit (Vazyme) was applied for qPCR to value target genes expression levels using 2^−ΔΔCt^ method. Primer sequences were showed in Supplementary Table 3 with GAPDH served as housekeeping gene.

### Western blotting (WB) and human apoptosis antibody array

Total protein was extracted using ice-cold RIPA buffer (Millipore) for further electrophoresis. In every WB analysis, equal amount protein was separated by SDS-polyacrylamide gel electrophoresis (PAGE) (10%, Invitrogen) and electro-transferred onto Poly-(vinylidene fluoride) membranes. Antibodies (Supplementary Table 1) were used to identify the target protein with corresponding second antibodies. For human apoptosis protein detection, the array membrane was blocked and then incubated with protein samples overnight at 4° C and proceeded to incubate with HRP linked streptavidin for another 1 h. Signals were captured by ECL-plus^TM^ WB system (Amersham) and protein gray images were analyzed by ImageJ.

### MTT and Celigo assay

Transfected HUCCT1 cells were seeded into a plate with 96 wells (2,000 cells per well). Cell viability and growth was measured using MTT solution (GenView). CD490 value were obtained to draw the cell growth curve (five days) using a microplate reader (Tecan). Live cells in five consecutive days were counted by Celigo image cytometer (Nexcelom Bioscience).

### Fluorescence activated cell sorting assay

Lentivirus transfected cells were collected for apoptosis and cycle assay detecting. Annexin V-APC (eBioscience) staining was used for cell apoptosis, and PI (BD Biosciences) staining was used for cell cycle. Cells were measured using FACS Calibur (BD Biosciences).

### Scratch assay

Transfected cells were grown to 90% confluence, and scratches across monolayer were made by a VP scientific wounding replicator. The migration distance was recorded for consecutive two days post scratching using ImageJ software.

### Colony formation assay

Transfected HUCCT1 cells were cultured in a plate with six well in triplicate. After ten days growing, 4% paraformaldehyde was added into each well for cells fixing, next, clones were washed and stained with Giemsa and fluorescence pictures was captured under an Olympus microscope. The colonies number was counted.

### RNA-sequencing

Following the manufacturer's instruction manual, total RNA in transfected HCCC-9810 cells was extracted and RNA concentration and quality was determined. Gene expression profile detection was accomplished using Affymetrix human GeneChip PrimeView gene expression array. Welch t-test and Benjamini-Hochberg FDR were used to assess raw data statistical significance (|Fold Change| ≥ 1.3 and FDR < 0.05 as significant). Next, significant data was used for Ingenuity Pathway Analysis (IPA) (Qiagen) executing (|Z - score| > 2 as significant).

### Xenograft mouse model

Female BALB/c nude mice (Beijing Vital River Laboratory Animal Technology Co., Ltd (Beijing, China)) were grown for xenograft mouse model experiments. 20 4-week-old mice were randomly divided into shCtrl group and shPSMC2 group and were injected subcutaneously with LV-shCtrl or LV-shPSMC2 transfected HUCCT1 cells, then housed in a 12/12 day/night cycle environment at 24° C with 60% humidity. The weight of mouse and the size of tumors were monitored. All mice were anesthetized used 0.7% Pentobarbital Sodium (10 μL/g) by intraperitoneal injection 67 days after the injection. The Berthold Technologies living imaging system was used to collect the *in vivo* bioluminescence images. Then all mice were sacrificed and tumors were harvested and the volumes were measured using a Vernier caliper. Mice tumor tissues were stained by Ki-67 staining assay. All animal studies were approved by Ethics committee of Hunan Provincial People’s Hospital.

### Statistical analyses

Data in triplicate are expressed as mean ± SD. Statistical significance (calculated by SPSS 22.0 (IBM) and Graphpad Software (GraphPad Prism 6.01)) was assessed at *P* < 0.05 and analyzed using Student’s t test or One-way ANOVA. Sign test was used to assess the difference of PSMC2 expression in cholangiocarcinoma tissues and normal tissues revealed in immunohistochemistry assay. Mann-Whitney U analysis and Spearman rank correlation analysis was used to analyze the relationships between PSMC2 expression and tumor characteristics.

## Supplementary Material

Supplementary Figures

Supplementary Tables

## References

[1] Razumilava N, Gores GJ. Cholangiocarcinoma. Lancet. 2014; 383:2168–79. 10.1016/S0140-6736(13)61903-0 24581682 PMC4069226

[2] Doherty B, Nambudiri VE, Palmer WC. Update on the Diagnosis and Treatment of Cholangiocarcinoma. Curr Gastroenterol Rep. 2017; 19:2. 10.1007/s11894-017-0542-4 28110453

[3] Oliveira IS, Kilcoyne A, Everett JM, Mino-Kenudson M, Harisinghani MG, Ganesan K. Cholangiocarcinoma: classification, diagnosis, staging, imaging features, and management. Abdom Radiol (NY). 2017; 42:1637–49. 10.1007/s00261-017-1094-7 28271275

[4] Rizvi S, Gores GJ. Pathogenesis, diagnosis, and management of cholangiocarcinoma. Gastroenterology. 2013; 145:1215–29. 10.1053/j.gastro.2013.10.013 24140396 PMC3862291

[5] Rizvi S, Khan SA, Hallemeier CL, Kelley RK, Gores GJ. Cholangiocarcinoma - evolving concepts and therapeutic strategies. Nat Rev Clin Oncol. 2018; 15:95–111. 10.1038/nrclinonc.2017.157 28994423 PMC5819599

[6] Lee YT, Tan YJ, Oon CE. Molecular targeted therapy: Treating cancer with specificity. Eur J Pharmacol. 2018; 834:188–96. 10.1016/j.ejphar.2018.07.034 30031797

[7] Kumar B, Singh S, Skvortsova I, Kumar V. Promising Targets in Anti-cancer Drug Development: Recent Updates. Curr Med Chem. 2017; 24:4729–52. 10.2174/0929867324666170331123648 28393696

[8] Mahipal A, Kommalapati A, Tella SH, Lim A, Kim R. Novel targeted treatment options for advanced cholangiocarcinoma. Expert Opin Investig Drugs. 2018; 27:709–20. 10.1080/13543784.2018.1512581 30124336

[9] Schweitzer N, Vogel A. Systemic therapy of cholangiocarcinoma: From chemotherapy to targeted therapies. Best Pract Res Clin Gastroenterol. 2015; 29:345–53. 10.1016/j.bpg.2015.01.002 25966433

[10] Chong DQ, Zhu AX. The landscape of targeted therapies for cholangiocarcinoma: current status and emerging targets. Oncotarget. 2016; 7:46750–67. 10.18632/oncotarget.8775 27102149 PMC5216834

[11] Zwergel T, Tahmatzopoulos A, Wullich B, Zwergel U, Stöckle M, Unteregger G. Proteasome inhibitors and their combination with antiandrogens: effects on apoptosis, cellular proliferation and viability of prostatic adenocarcinoma cell cultures. Prostate Cancer Prostatic Dis. 2004; 7:138–43. 10.1038/sj.pcan.4500709 15069423

[12] da Fonseca PC, He J, Morris EP. Molecular model of the human 26S proteasome. Mol Cell. 2012; 46:54–66. 10.1016/j.molcel.2012.03.026 22500737

[13] Wakshlag JJ, Kallfelz FA, Barr SC, Ordway G, Haley NJ, Flaherty CE, Kelley RL, Altom EK, Lepine AJ, Davenport GM. Effects of exercise on canine skeletal muscle proteolysis: an investigation of the ubiquitin-proteasome pathway and other metabolic markers. Vet Ther. 2002; 3:215–25. 12447828

[14] Tanahashi N, Suzuki M, Fujiwara T, Takahashi E, Shimbara N, Chung CH, Tanaka K. Chromosomal localization and immunological analysis of a family of human 26S proteasomal ATPases. Biochem Biophys Res Commun. 1998; 243:229–32. 10.1006/bbrc.1997.7892 9473509

[15] Smith DM, Fraga H, Reis C, Kafri G, Goldberg AL. ATP binds to proteasomal ATPases in pairs with distinct functional effects, implying an ordered reaction cycle. Cell. 2011; 144:526–38. 10.1016/j.cell.2011.02.005 21335235 PMC3063399

[16] Nijhawan D, Zack TI, Ren Y, Strickland MR, Lamothe R, Schumacher SE, Tsherniak A, Besche HC, Rosenbluh J, Shehata S, Cowley GS, Weir BA, Goldberg AL, et al. Cancer vulnerabilities unveiled by genomic loss. Cell. 2012; 150:842–54. 10.1016/j.cell.2012.07.023 22901813 PMC3429351

[17] Deshpande R, Asiedu MK, Klebig M, Sutor S, Kuzmin E, Nelson J, Piotrowski J, Shin SH, Yoshida M, Costanzo M, Boone C, Wigle DA, Myers CL. A comparative genomic approach for identifying synthetic lethal interactions in human cancer. Cancer Res. 2013; 73:6128–36. 10.1158/0008-5472.CAN-12-3956 23980094 PMC3809957

[18] He J, Xing J, Yang X, Zhang C, Zhang Y, Wang H, Xu X, Wang H, Cao Y, Xu H, Zhang C, Wang C, Yu E. Silencing of Proteasome 26S Subunit ATPase 2 Regulates Colorectal Cancer Cell Proliferation, Apoptosis, and Migration. Chemotherapy. 2019; 64:146–54. 10.1159/000502224 31715603

[19] Saeki Y. Ubiquitin recognition by the proteasome. J Biochem. 2017; 161:113–24. 10.1093/jb/mvw091 28069863

[20] Bard JA, Goodall EA, Greene ER, Jonsson E, Dong KC, Martin A. Structure and Function of the 26S Proteasome. Annu Rev Biochem. 2018; 87:697–724. 10.1146/annurev-biochem-062917-011931 29652515 PMC6422034

[21] Motegi A, Murakawa Y, Takeda S. The vital link between the ubiquitin-proteasome pathway and DNA repair: impact on cancer therapy. Cancer Lett. 2009; 283:1–9. 10.1016/j.canlet.2008.12.030 19201084

[22] Song M, Wang Y, Zhang Z, Wang S. PSMC2 is up-regulated in osteosarcoma and regulates osteosarcoma cell proliferation, apoptosis and migration. Oncotarget. 2017; 8:933–53. 10.18632/oncotarget.13511 27888613 PMC5352207

[23] Li GW, Yan X. Lower miR-630 expression predicts poor prognosis of osteosarcoma and promotes cell proliferation, migration and invasion by targeting PSMC2. Eur Rev Med Pharmacol Sci. 2019; 23:1915–25. 10.26355/eurrev_201903_17229 30915734

[24] Adams JM, Cory S. The Bcl-2 protein family: arbiters of cell survival. Science. 1998; 281:1322–26. 10.1126/science.281.5381.1322 9735050

[25] Leibowitz B, Yu J. Mitochondrial signaling in cell death via the Bcl-2 family. Cancer Biol Ther. 2010; 9:417–22. 10.4161/cbt.9.6.11392 20190564 PMC2874116

[26] Gahl RF, Dwivedi P, Tjandra N. Bcl-2 proteins bid and bax form a network to permeabilize the mitochondria at the onset of apoptosis. Cell Death Dis. 2016; 7:e2424. 10.1038/cddis.2016.320 27763642 PMC5133987

[27] Fornari F, Pollutri D, Patrizi C, La Bella T, Marinelli S, Casadei Gardini A, Marisi G, Baron Toaldo M, Baglioni M, Salvatore V, Callegari E, Baldassarre M, Galassi M, et al. In Hepatocellular Carcinoma miR-221 Modulates Sorafenib Resistance through Inhibition of Caspase-3-Mediated Apoptosis. Clin Cancer Res. 2017; 23:3953–65. 10.1158/1078-0432.CCR-16-1464 28096271

[28] Goldar S, Khaniani MS, Derakhshan SM, Baradaran B. Molecular mechanisms of apoptosis and roles in cancer development and treatment. Asian Pac J Cancer Prev. 2015; 16:2129–44. 10.7314/apjcp.2015.16.6.2129 25824729

[29] Philchenkov A, Miura K. The IAP Protein Family, SMAC Mimetics and Cancer Treatment. Crit Rev Oncog. 2016; 21:185–202. 10.1615/CritRevOncog.2016017032 27915971

[30] Martínez-García D, Manero-Rupérez N, Quesada R, Korrodi-Gregório L, Soto-Cerrato V. Therapeutic strategies involving survivin inhibition in cancer. Med Res Rev. 2019; 39:887–909. 10.1002/med.21547 30421440

[31] Chen T, You Y, Jiang H, Wang ZZ. Epithelial-mesenchymal transition (EMT): A biological process in the development, stem cell differentiation, and tumorigenesis. J Cell Physiol. 2017; 232:3261–72. 10.1002/jcp.25797 28079253 PMC5507753

[32] Liao TT, Yang MH. Revisiting epithelial-mesenchymal transition in cancer metastasis: the connection between epithelial plasticity and stemness. Mol Oncol. 2017; 11:792–804. 10.1002/1878-0261.12096 28649800 PMC5496497

[33] Serrano-Gomez SJ, Maziveyi M, Alahari SK. Regulation of epithelial-mesenchymal transition through epigenetic and post-translational modifications. Mol Cancer. 2016; 15:18. 10.1186/s12943-016-0502-x 26905733 PMC4765192

[34] Meng J, Chen S, Han JX, Qian B, Wang XR, Zhong WL, Qin Y, Zhang H, Gao WF, Lei YY, Yang W, Yang L, Zhang C, et al. Twist1 Regulates Vimentin through Cul2 Circular RNA to Promote EMT in Hepatocellular Carcinoma. Cancer Res. 2018; 78:4150–62. 10.1158/0008-5472.CAN-17-3009 29844124

[35] Liao H, Ji F, Ying S. CDK1: beyond cell cycle regulation. Aging (Albany NY). 2017; 9:2465–66. 10.18632/aging.101348 29242409 PMC5764383

[36] Ravindran Menon D, Luo Y, Arcaroli JJ, Liu S, KrishnanKutty LN, Osborne DG, Li Y, Samson JM, Bagby S, Tan AC, Robinson WA, Messersmith WA, Fujita M. CDK1 Interacts with Sox2 and Promotes Tumor Initiation in Human Melanoma. Cancer Res. 2018; 78:6561–74. 10.1158/0008-5472.CAN-18-0330 30297536 PMC6279496

[37] Tian Z, Cao S, Li C, Xu M, Wei H, Yang H, Sun Q, Ren Q, Zhang L. LncRNA PVT1 regulates growth, migration, and invasion of bladder cancer by miR-31/ CDK1. J Cell Physiol. 2019; 234:4799–811. 10.1002/jcp.27279 30317572

[38] Wattanawongdon W, Hahnvajanawong C, Namwat N, Kanchanawat S, Boonmars T, Jearanaikoon P, Leelayuwat C, Techasen A, Seubwai W. Establishment and characterization of gemcitabine-resistant human cholangiocarcinoma cell lines with multidrug resistance and enhanced invasiveness. Int J Oncol. 2015; 47:398–410. 10.3892/ijo.2015.3019 25998688

