# Supplementary material for: Knockdown of PSMC2 contributes to suppression of cholangiocarcinoma development by regulating CDK1
Source: Aging (Albany NY). 2021 Sep 9;13(17):21325–44. doi: 10.18632/aging.203463 (PMC8457563; doi:10.18632/aging.203463)
Supplement: Supplementary Figures [file aging-13-203463-s001.pdf]

SUPPLEMENTARY FIGURES

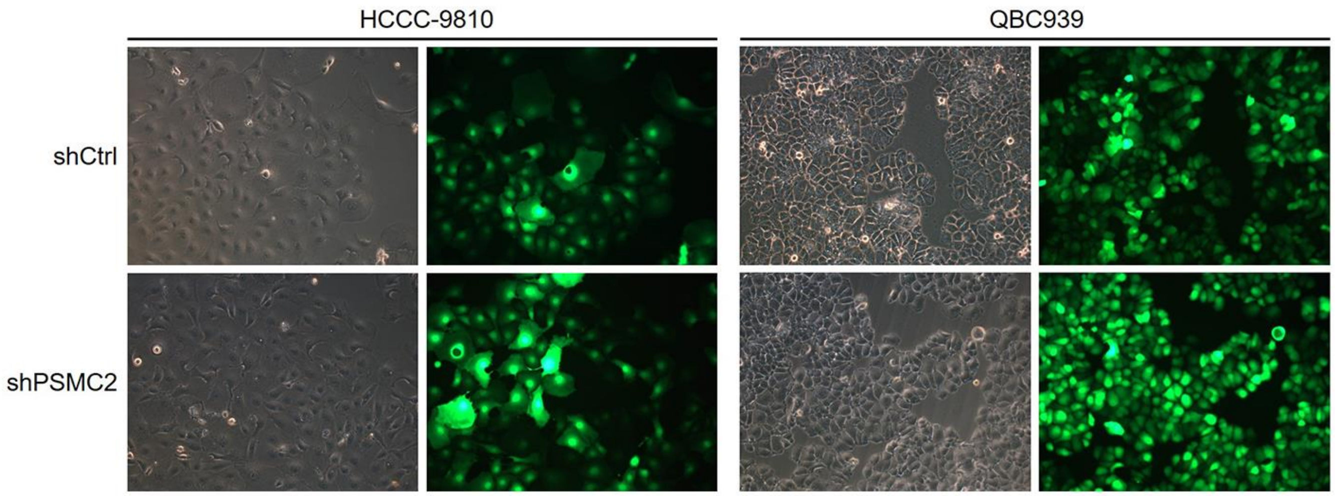

Supplementary Figure 1. The transfection efficiencies of shPSMC2 and shCtrl in HCCC-9810 and QBC939 cells were evaluated through observing the fluorescence of GFP on lentivirus vector.

A

B

C

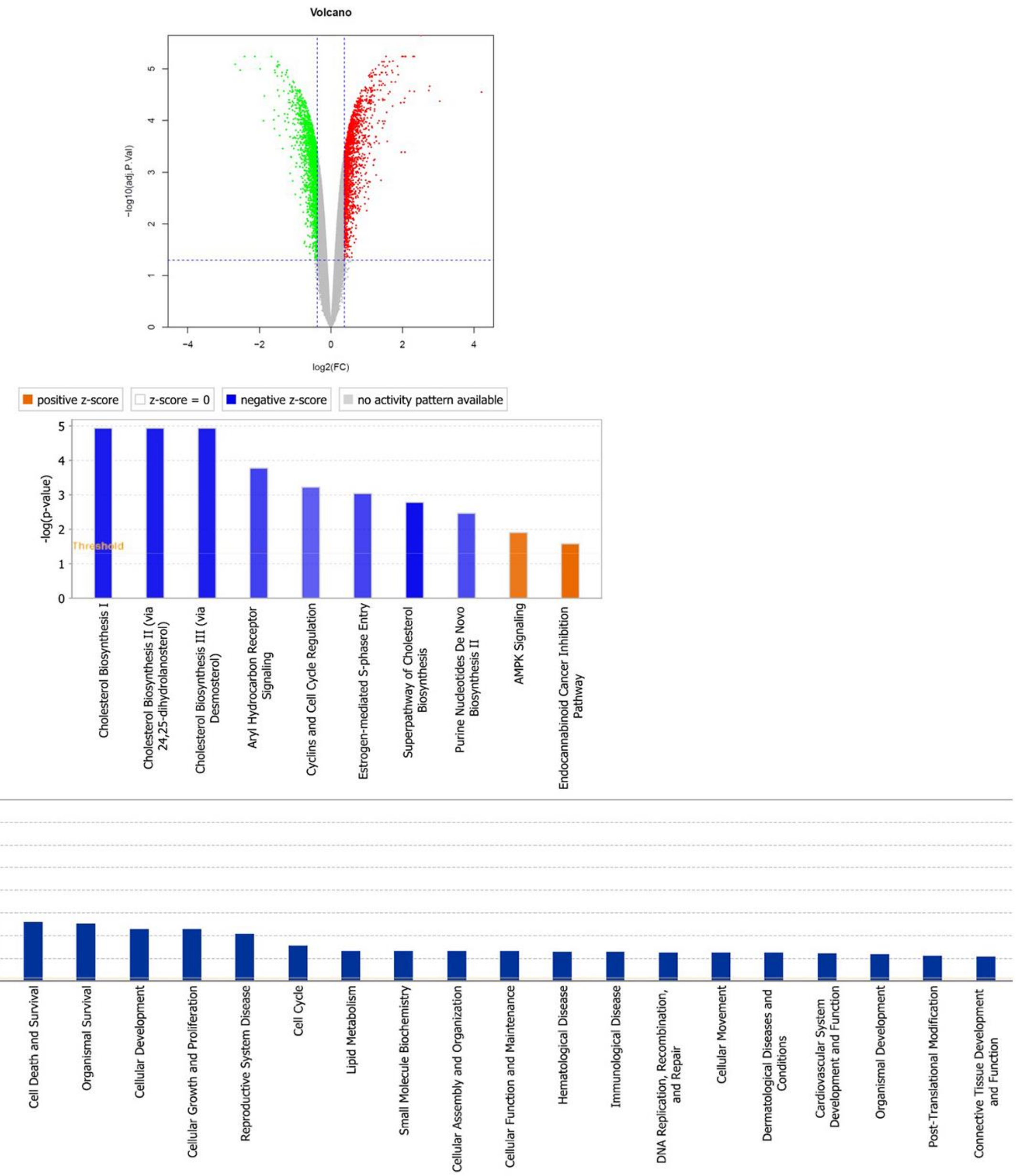

Supplementary Figure 2. (**A**) The volcano plot of gene expression profiling in HCCC-9810 cells with or without PSMC2 knockdown. Red dots represented significantly upregulated DEGs. Green dots represented significantly downregulated DEGs. (**B**) The enrichment of the DEGs in canonical signaling pathways was analyzed by IPA. (**C**) The enrichment of the DEGs in IPA disease and function was analyzed by IPA. Data was shown as mean ± SD. **P* < 0.05, ***P* < 0.01, ****P* < 0.001.

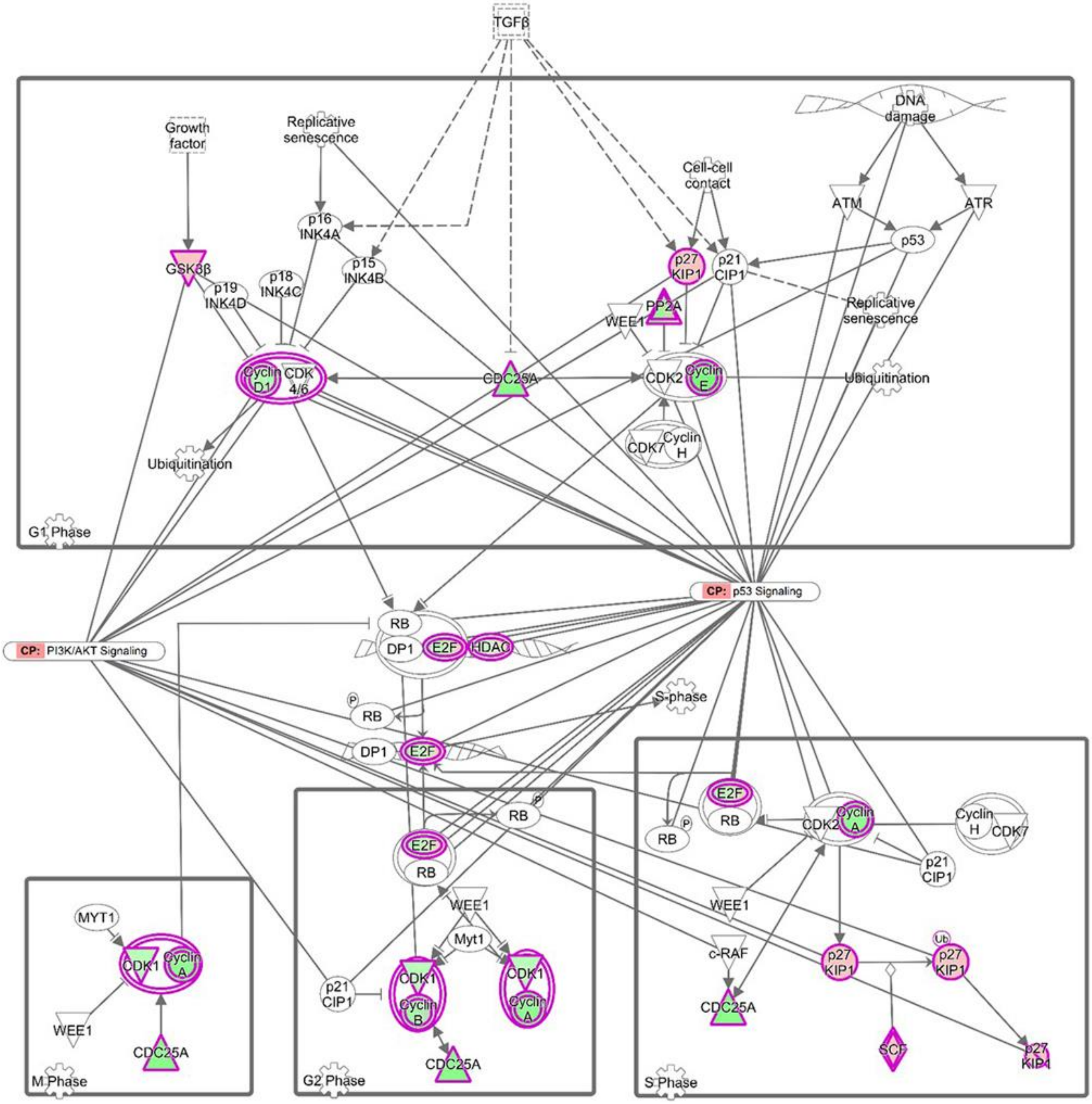

Supplementary Figure 3. The histogram of cyclin and cell cycle regulation pathway.

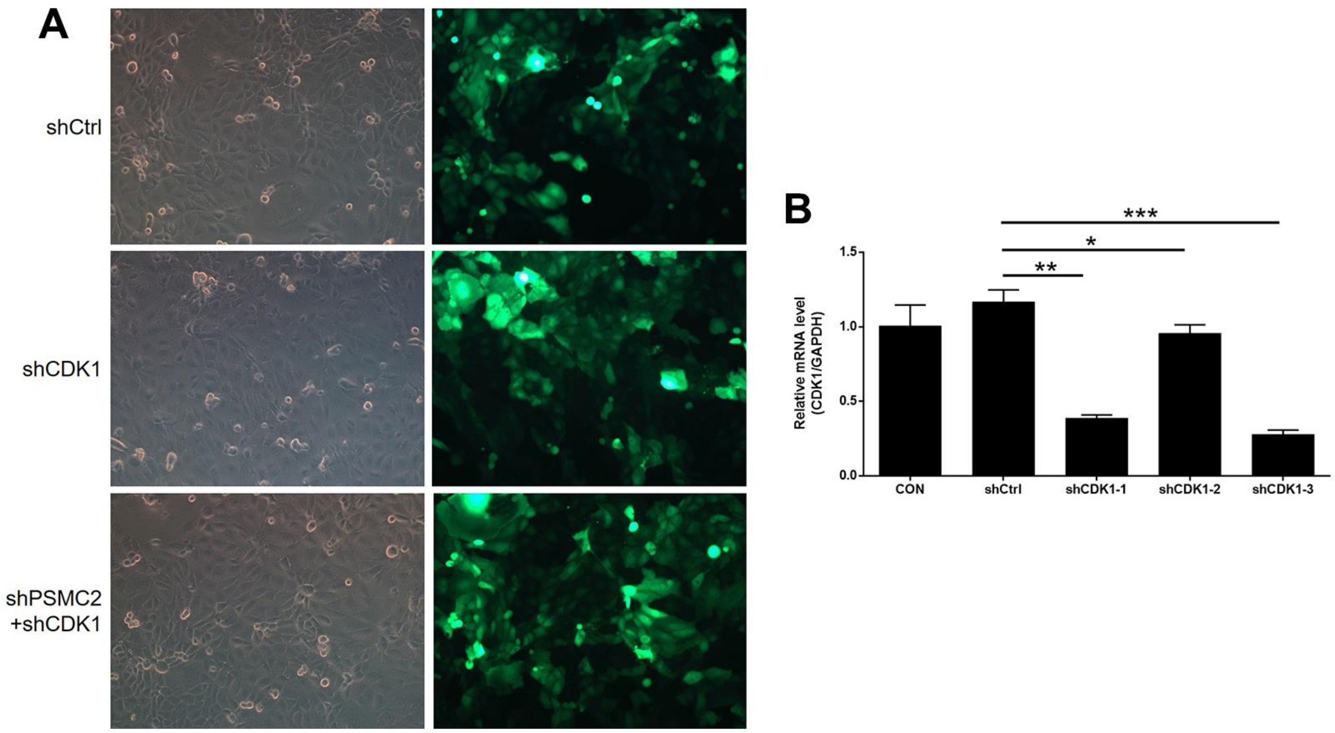

Supplementary Figure 4. (**A**) The transfection efficiencies of shCtrl, shCDK1, shPSMC2+shCDK1 in HCCC-9810 cells were evaluated through observing the fluorescence of GFP on lentivirus vector. (**B**) The knockdown efficiencies of 3 shRNAs designed for CDK1 knockdown were evaluated by qPCR in HCCC-9810 cells. Data was shown as mean ± SD. **P* < 0.05, ***P* < 0.01, ****P* < 0.001.

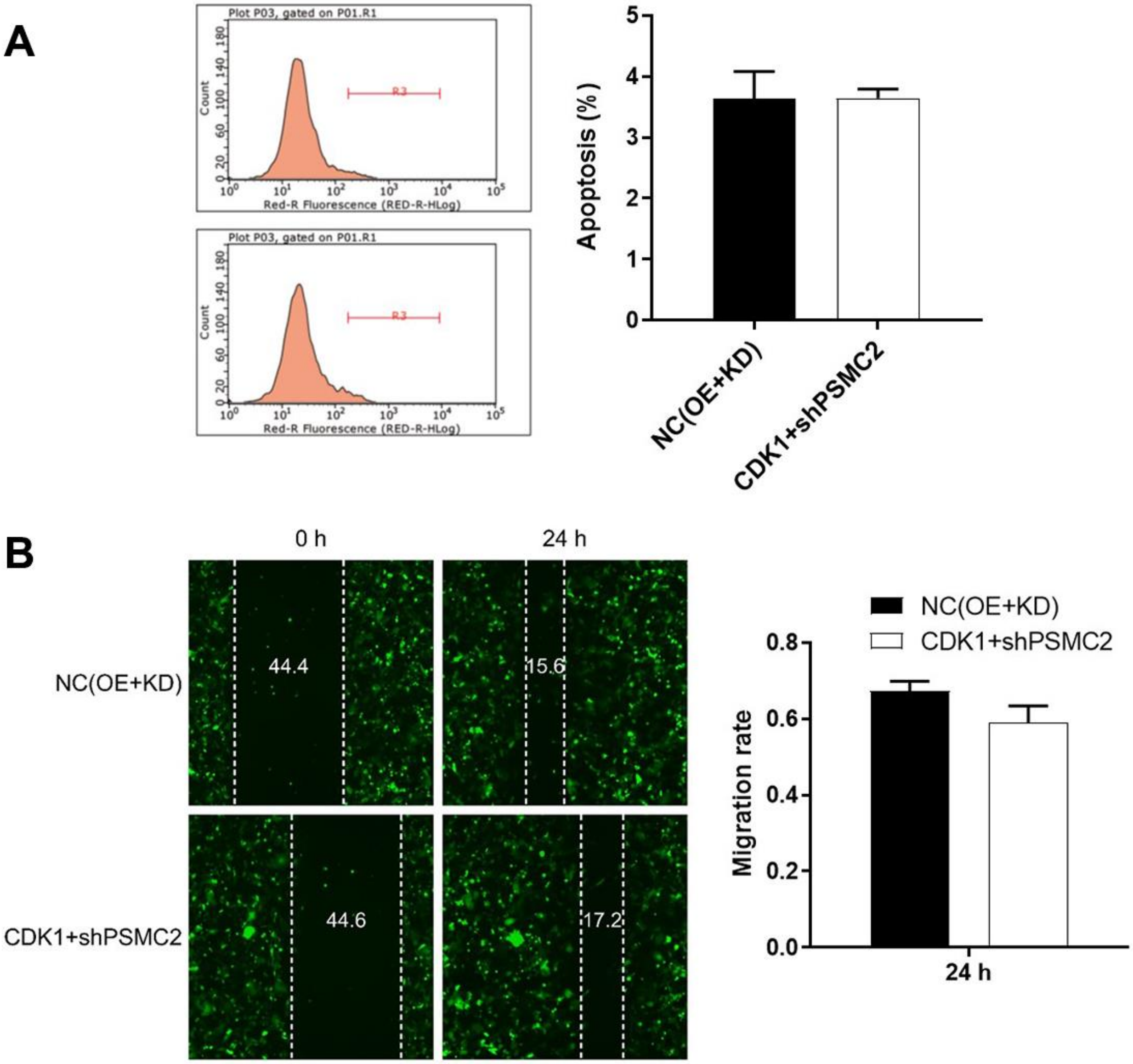

Supplementary Figure 5. (**A**) Flow cytometry was performed to detect the apoptosis of CCA cells with simultaneous CDK1 overexpression and PSMC2 knockdown. (**B**) Wound-healing assay was carried out to assess cell migration of CCA cells with simultaneous CDK1 overexpression and PSMC2 knockdown. Data was shown as mean ± SD.
