# Supplementary material for: Knockdown of PSMC2 contributes to suppression of cholangiocarcinoma development by regulating CDK1
Source: Aging (Albany NY). 2021 Sep 9;13(17):21325–44. doi: 10.18632/aging.203463 (PMC8457563; doi:10.18632/aging.203463)
Supplement: Supplementary Tables [file aging-13-203463-s002.pdf]

SUPPLEMENTARY TABLES

Supplementary Table 1. Antibodies used in western blotting and IHC.

Primary antibodies	Dilution in WB	Source species	Company	Catalog No.
PSMC2	1:1000	Mouse	Santa Cruz	SC-166972
GAPDH	1:3000	Rabbit	Bioworld	AP0063
E-cadherin	1:1000	Rabbit	CST	3195
N-cadherin	1:1000	Rabbit	abcam	ab18203
Vimentin	1:1000	Rabbit	abcam	ab92547
CCND1	1:2000	Rabbit	CST	2978
CDK1	1:2000	Rabbit	abcam	ab133327
E2F1	1:1500	Rabbit	abcam	ab179445
HDAC2	1:1000	Rabbit	abcam	ab32117
Cyclin B1	1:3000	Rabbit	abcam	ab32053
**Primary antibodies**	**Dilution in IHC**	**Source species**	**Company**	**Catalog No.**
PSMC2	1:50	Mouse	Santa Cruz	SC-166972
Ki67	1:200	Rabbit	abcam	ab16667
CDK1	1:100	Rabbit	abcam	ab133327
**Secondary antibody**	**Dilution**		**Company**	**Catalog No.**
HRP Goat Anti-Rabbit IgG (WB)	1:3000		Beyotime	A0208
HRP Goat Anti-Mouse IgG (WB)	1:3000		Beyotime	A0216
HRP Goat Anti-Mouse IgG (IHC)	1:2000		abcam	ab205719
HRP Goat Anti-Rabbit IgG (IHC)	1:400		abcam	Ab6721

Supplementary Table 2. The target sequences and shRNA sequences.

Gene	No.	Target sequence (5'-3')	shRNA sequences (5'-3')
PSMC2	Human-PSMC2-1	GCCAGGGAGATTGGATAGAAA	ccggGCCAGGGAGATTGGATAGAAAttcaagagaTTTCTATCCAATCTCCCTGGCtttttg<br>aattcaaaaaGCCAGGGAGATTGGATAGAAAttcaagaga
CDK1	Human-CDK1-1	TTCCATGGATCTGAAGAAATA	CcggTTCCATGGATCTGAAGAAATActcgagTATTTCTTCAGATCCATGGAATTTTTg<br>aattcaaaaaTTCCATGGATCTGAAGAAATActcgagTATTTCTTCAGATCCATGGAA
CDK1	Human-CDK1-2	AGACTAGAAAGTGAAGAGGAA	CcggAGACTAGAAAGTGAAGAGGAActcgagTTCCTCTTCACTTTCTAGTCTTTTTTg<br>aattcaaaaaAGACTAGAAAGTGAAGAGGAActcgagTTCCTCTTCACTTTCTAGTCT
CDK1	Human-CDK1-3	ATGGAGTTGTGTATAAGGGTA	CcggATGGAGTTGTGTATAAGGGTActcgagTACCCTTATACACAACTCCATTTTTg<br>aattcaaaaaATGGAGTTGTGTATAAGGGTActcgagTACCCTTATACACAACTCCAT

Supplementary Table 3. Primers used in qPCR.

Gene	Forward primer sequence (5'-3')	Reverse primer sequence (5'-3')
GAPDH	TGACTTCAACAGCGACACCCA	CACCCTGTTGCTGTAGCCAAA
PSMC2	CAGCACTCTGGGATTTGGCT	TTTCTATCCACGCCCACTCTC
HDAC2	TCTATTCGAGCATCAGACAAGC	GCCACATTTCTTCGACCTCCT
ALDH18A1	CAGGTGGCAGCTTTGGCTAT	TGGGTCAGGAGGTGGAGAAT
HDAC9	GCTGGTGGAGTTCCCTTACAT	AAAGGTGCAGACTGGGTTCG
ALDH1A1	GTCAAACCAGCAGAGCAAACT	TTCACTACTCCAGGAGGAAACC
MAP3K5	GTCGGGACTTGAGCAACCAA	TCACTGAAAGAGCCCAGATACTG
ALDH1A3	GGTTAAAGAAGCTGCGTCCC	GGTTGAAGAACACTCCCTGATG
MCM7	ATCACGGTGCTGGTAGAAGG	CGCAGGATTGGCAAGAAA
BIRC5	TCTCAAGGACCACCGCATCT	TTTGCATGGGGTCGTCATCT
NFIB	CTTATCCAATCCCGACCAGA	GACTAGATCCAGACGCCAGACT
BRCA1	TGGCAACATACCATCTTCAACC	TGTCAATTCTGGCTTCTCCCT
CCNA2	AGCCTGCGTTCACCATTCA	GGGCATCTTCACGCTCTATTTT
CCNB2	AAGTTCCAGTTCAACCCACCA	GCAGAGCAAGGCATCAGAAAA
SMC1A	TCTTGCCTCTTGACTACCTGG	TGGCTCATAGCGAATCACATC
CCND1	AGCTGTGCATCTACACCGAC	GAAATCGTGCGGGGTCATTG
STAT3	GATTGACCAGCAGTATAGCCG	GCAGTCTGTAGAAGGCGTGAT
CDC25A	TGGAAGTACAAAGAGGAGGAAGAG	GCCAGGGATAAAGACTGATGAAG
UBE2C	AAGTTCCTCACGCCCTGCTAT	TGGCTGGTGACCTGCTTTGA
CCNB1	AAACTTTGGTCTGGGTCGGC	TGCTGCAATTTGAGAAGGAGG
E2F1	CACTTTCGGCCCTTTTGCTC	GTGCTCTCACCGTCCTACAC
FOS	CAGACTACGAGGCGTCATCC	TCTGCGGGTGAGTGGTAGTA

Supplementary Table 4. Relationship between PSMC2 expression and tumor characteristics in patients with cholangiocarcinoma analyzed by spearman rank correlation analysis.

Tumor characteristics	Index	
Grade	Pearson correlation	0.624
	Significance (two tailed)	<0.001
	n	70
